# Impact of distribution campaigns on the use and availability of long-lasting insecticide-treated nets in rural Guinea

**DOI:** 10.5281/zenodo.20429788

**Published:** 2026-05-28

**Authors:** Emre Toner, Benjamin Plato, Abbyomi Johnson, Afi Dobson, Amanda Jackson, Caroline Read, Dirk Chisholm, Hawa Jabbie, Abdoulaye Barry

**Affiliations:** 1 Peace Corps Guinea, B.P. 1927, Conakry, Guinea.

## Abstract

**Background:**

Despite ongoing efforts by both Guinean authorities and international stakeholders, malaria remains the leading public health challenge in Guinea, contributing to significant morbidity and mortality, especially among children under five. This study aimed to evaluate the effectiveness of distribution campaigns in increasing ownership and utilisation of long-lasting insecticide-treated nets (LLINs), to improve coverage among this high-risk population.

**Materials and Methods:**

A cross-sectional, community-based survey of 34 villages in Guinea (May–August 2025) assessed household LLIN ownership and use. A total of 316 household data records were collected door-to-door by Peace Corps Volunteers and trained local health workers using Google Forms or printed surveys in local languages. Households with and without recent LLIN distribution campaigns were compared using non-parametric Mann–Whitney U tests.

**Results:**

Campaign presence was associated with increased household ownership of at least one LLIN, from 45.8% to 94.5% (p < 0.0001). Mean population access to LLINs saw a three-fold increase from 20.8% to 72.9% (p < 0.001). LLIN use among children under five doubled from 24.8% to 50.0% (p < 0.001), while adult use rose from 28.3% to 42.0% (p = 0.052) in campaign-exposed areas. At the household level, net density reached the target of 1.00 net per sleeping space (up from 0.00; p < 0.001). While median ownership reached 1.00 net per bed, the median number of nets actually hung was only 0.55, indicating that logistical success has not yet translated into universal deployment.

**Conclusions:**

The LLIN distribution campaign was associated with substantially increased bednet ownership, population access, and use in Guinea, underscoring the critical role of such efforts by the Ministry of Health and international partners. However, underutilisation persists due to social, cultural, and practical barriers. Strengthening post-distribution support and targeted sensitisation campaigns is essential to ensure proper use and maximise protection for vulnerable populations.

## Introduction

Despite ongoing efforts by the Guinean Ministry of Health and international stakeholders, malaria remains a critical national health concern. Malaria transmission in Guinea peaks between July and October, coinciding with the country’s rainy season. Approximately 92% of malaria cases are caused by *Plasmodium falciparum*, the most virulent of the malaria parasites found in sub-Saharan Africa. According to data from the President’s Malaria Initiative (PMI), malaria accounts for about 31% of medical consultations, 25% of hospital admissions, and 14% of hospital deaths among children under five years of age in public facilities. These figures exclude cases managed in community and private healthcare settings, suggesting that the overall burden is likely even higher across the population [1].

Long-Lasting Insecticidal Nets (LLINs) represent a critical advancement in vector control, remaining effective for at least three years through factory-integrated insecticide treatment. Their efficacy in reducing malaria morbidity and all-cause child mortality is well established across diverse endemic settings [2-5]. Specifically, systematic reviews indicate a reduction in child mortality from 33 to 27 per 1,000 person-years [2], while large-scale analyses of DHS data suggest a 21% reduction in the odds of infection, with maximal protection observed in nets less than one year old [3].

The practical success of LLINs depends on the ability of mass distribution campaigns to bridge the gap between household ownership and consistent individual use. Evidence from large-scale campaigns in Uganda and the Democratic Republic of Congo (DRC) demonstrates that while free distribution can rapidly increase ownership rates to over 90%, individual usage often lags behind access [6-9]. Furthermore, the longitudinal decline in usage observed by Okiring *et al.* [10] highlights the necessity of time-sensitive, granular assessments to evaluate the sustained impact of these campaigns.

Since 2011, the U.S. President’s Malaria Initiative (PMI) and the Global Fund, in collaboration with Catholic Relief Services (CRS) and other partners, have aimed to distribute bednets, train community healthcare workers, and educate citizens across Guinea. Nets are procured for both routine and mass campaign distributions, although stockouts have persisted in some areas. Local associations, governments, and health clinics play a key role in planning and delivering nets, including translating campaign information into local languages, while health centre staff and community healthcare workers provide household-level support to ensure effective net use. Health centres and posts are also expected to provide LLINs free of charge to pregnant women and families with children under five, ensuring vulnerable populations have access to preventive tools. Nevertheless, practical barriers to LLIN access and consistent usage remain [11].

Although mass LLIN distribution campaigns have substantially increased household ownership across malaria-endemic settings, persistent gaps between ownership and use suggest that coverage alone may be insufficient to sustain protection. Evidence indicates that the assumption underpinning standard three-year replacement cycles may not reflect real-world conditions: physical wear, washing frequency, and environmental exposures such as indoor cooking smoke can shorten the functional lifespan of LLINs, contributing to early attrition [12]. At the same time, qualitative studies suggest that use is shaped by behavioural and contextual factors, including thermal discomfort, seasonal perceptions of mosquito risk, and irregular sleeping arrangements, which may reduce consistent utilisation despite availability [13-14]. These findings raise broader questions about the limitations of mass campaigns as a standalone strategy, particularly in remote rural settings where replacement access and sustained adherence may be constrained. Understanding how durability-related attrition and behavioural factors interact in such contexts is therefore important for explaining persistent ownership–use gaps and improving malaria prevention efforts.

**Figure 1:**
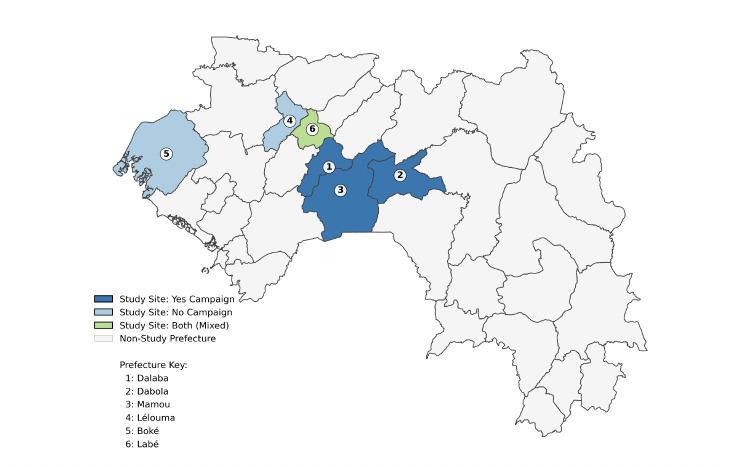
Geographic distribution of study prefectures in Guinea stratified by campaign status. Prefectures involved in the household surveys are coloured by campaign exposure: dark blue ("Yes Campaign"), light blue ("No Campaign"), and light green ("Both"). Non-study prefectures are shown in light grey. Administrative boundaries sourced from GADM v4.1.

Most national malaria data in Guinea, including the Demographic and Health Surveys (DHS) and PMI surveys, are collected at the regional or prefecture level, often through centralised or semi-urban health posts [1,15]. Remote rural villages are often underrepresented due to access challenges, language barriers, and logistical constraints. To address this critical gap, we conducted a cross-sectional household survey across six prefectures in the Republic of Guinea (Figure 1), evaluating household-level ownership, usage patterns, and barriers to LLIN utilisation across remote regions of Guinea through a network of community-integrated Peace Corps volunteers, leveraging the longitudinal community presence of volunteers to minimise social desirability bias and overcome language barriers inherent in external surveying. The findings provide detailed insights to inform strategies for optimising distribution campaigns and improving protection for vulnerable populations. Data were collected in a cross-sectional manner throughout campaign areas and non-campaign areas of the 2025 national LLIN campaign. This campaign was modeled on a fixed-point strategy in which all residents in a district were given coupons to obtain their LLINs from an established distribution centre. Beyond quantitative outcomes, the study also documents field observations, such as cultural beliefs and logistical challenges, that are often overlooked in large-scale surveys.

LLIN coverage was examined through a selection of widely adopted standard indicators developed by the Roll Back Malaria (RBM) Monitoring and Evaluation Reference Group (MERG). Household ownership is defined as the proportion of households that own at least one LLIN. The Household Access Ratio is a measure of resource saturation at the domestic level, calculated as the number of available LLINs divided by the required number (assuming one net per two residents), with the ratio per household capped at 1.0. Conversely, Population Access represents the proportion of the total population that could potentially be covered by existing nets, calculated by multiplying the number of LLINs in households by two and dividing by the total number of residents. While the former assesses the success of the distribution supply chain, the latter reflects the actual protective potential for individuals. The World Health Organization (WHO) and Roll Back Malaria (RBM) Partnership have historically defined universal coverage as at least 80% of the population having access to an LLIN. LLIN use is defined as the number of household residents who slept under a LLIN the previous night [16-18].

## Methods

### Study design

The study adapted a survey previously used by U.S. Peace Corps health volunteers, adding questions about household bednet use and availability, as well as household demographics, to better assess the correlation between socioeconomic factors and the adoption of mosquito net use. The Google Forms version consisted of 58 questions, divided into 11 sections. The printable version, designed for use in communities with limited internet connectivity, consisted of 39 questions, followed by the final questions from the online version, which were related to the total bednet count in the household and converted into a table and tally sheet.

### Participants, locations, and timing

Seven volunteers with the U.S. Peace Corps (six health volunteers and one education volunteer) trained and worked alongside 40 Host Country Nationals (HCNs) selected from their host communities. This study was conducted in six prefectures in Guinea: Boké, Dabola, Dalaba, Labé, Lélouma, and Mamou (Figure 1). The study area map was generated using Python(v3.10) with the GeoPandas and Matplotlib libraries. Administrative boundaries for the Republic of Guinea were obtained from the Database of Global Administrative Areas (GADM, version 4.1) at the prefecture level (Administrative Level 2)[19]. Data were collected in a door-to-door manner from May 6, 2025, through August 14, 2025, in collaboration with local healthcare workers, coinciding with the regional rainy season. Households were selected through convenience sampling, ensuring approximately equal representation from villages that had recently received LLIN distributions and those that had not.

To capture a broad range of community dynamics, a multi-site convenience sampling strategy was employed across a total of 34 villages, with 128 households in campaign areas and 190 households in non-campaign areas. This approach was structured to ensure approximately equal representation from villages that had recently received LLIN distributions and those that had not. Within each village, households were approached sequentially based on real-time accessibility. A minimum two-week interval was maintained between the end of the campaign and the surveys to ensure households had sufficient time to hang their nets.

This specific post-intervention window was selected to capture the immediate operational uptake and initial ‘hang-up’ success of the campaign before confounding variables (e.g., mechanical net degradation, attrition, or long-term behavioural fatigue) altered usage behaviours. While large-scale national evaluations like the Demographic and Health Surveys (DHS) or Malaria Indicator Surveys (MIS) are traditionally timed to align with seasonal peak transmission or long-term steady states, our short-term window allowed for a targeted assessment of immediate behavioural mobilisation and net deployment efficacy at the community level.

### Training and preparation

Participating Peace Corps Volunteers (PCVs) joined a virtual training meeting to review and discuss the survey questions and survey campaign protocols. Volunteers were given links to the Google Forms survey and a printable version. PCVs then held in-person training within their communities for HCN participants in French, Pular, Maninka, and Susu. HCNs working in low-connectivity areas were given hard copies of the survey, which were later entered into the Google Forms document.

### Data collection

The majority of responses were collected directly into Google Forms, with roughly a quarter of responses being collected using physical copies and entered into Google Forms afterwards. As Google Forms did not allow for the recording of more than 6 LLINs/household, surveyors were instructed to enter separate responses for each building in a shared compound.

### Metrics

Households were assessed for their total bednet use rate, the use rate between genders, and the use rate for children under 5. Households were also questioned about the source of LLINs, which included being distributed via the 2025 LLIN campaign or previous campaigns, provided by the Health Centre during pre- and postnatal consultations (one per consultation), gifted, or purchased through local/regional markets.

### Data cleaning and preprocessing

To ensure data integrity and the robustness of the comparative analysis, a multi-stage logical consistency check was performed. First, extreme outliers were identified as observations exceeding two standard deviations from their respective group means. These outliers were individually assessed and determined to represent logistical implausibilities, such as reports of 20 bednets for a household of two, likely resulting from entry errors during field data collection via Google Forms. These records were excluded to mitigate skewing of availability metrics.

Second, a logical consistency check was applied to utilisation data to ensure the integrity of coverage proportions. Households were excluded if the reported number of residents (either children under five or adults) who slept under a bednet the previous night exceeded the total reported number of residents in that category for the household. In this study, 75 households were excluded due to such discrepancies (38 non-campaign and 37 campaign). These inconsistencies likely reflect the fluid and transient nature of rural Guinean households, where members of extended families frequently relocate or ‘visit’ different households, particularly during school or agricultural cycles. By applying this strict exclusion criterion, we avoided the introduction of artificial inflation and ensured a conservative, representative estimate of typical household utilisation. After rigorous data cleaning, the final analytical sample consisted of 181 unique households, including 115 in non-campaign areas and 66 in campaign-exposed areas.

### Statistical analysis

Data processing and analysis were performed using Python 3 (v3.10) with the pandas and matplotlib libraries. Normality of the distributions was assessed separately within groups (presence vs. absence of bednet distribution campaigns) using the Shapiro–Wilk test. The test indicated non-normal distributions in both groups (non-campaign group: W = 0.71, p < 0.001; campaign group: W = 0.94, p = 0.005). Consequently, non-parametric testing using the Mann–Whitney U test was applied to compare outcome variables between the groups. Asterisks in the figures indicate statistical significance: one asterisk (*) for p < 0.05, two asterisks (**) for p < 0.01, and three asterisks (***) for p < 0.001.

### Ethical clearance and consent

Verbal informed consent was obtained from all participating households prior to data collection. Participation was voluntary, and respondents were informed of the study’s purpose and assured of confidentiality. No personally identifiable information was collected. The study protocol was reviewed and approved by the U.S. Peace Corps Guinea office.

## Results

### Impact of distribution campaign on LLIN indicators

The primary LLIN indicators regarding bednet ownership, access, and use are summarised in Table 1. The data demonstrates a substantial increase in all coverage metrics associated with the distribution campaign. Household ownership of at least one LLIN reached 94.5% in campaign areas, while population access—defined as the proportion of individuals with access to an LLIN within the household—was 72.9% (Table 1). Despite these observed gains in access, individual use remained lower, particularly among the adult population, suggesting a persistent gap between availability and use.

**Table 1:** Summary of LLIN indicators by campaign status (SD = Standard deviation; SEM = Standard error of the mean.

Indicator	No Campaign (n=153)	Yes Campaign (n=103)	p-value
Household Characteristics			
Mean household size (SD)	5.8 (2.1)	6.1 (2.4)	0.312
LLIN Ownership and Access			
Households with at least one LLIN (%)	46.4	94.2	< 0.001
Population access (%)	23.4	77.8	< 0.001
LLIN Utilization			
Analytical sub-sample size (n)	115	66	
Children under 5 (% ± SEM)	24.8 ± 3.6	50.0 ± 6.0	0.0009
Adults (% ± SEM)	28.3 ± 3.5	42.0 ±5.5	0.052
Net Condition and Sources			
Mean nets per household	0.92	2.84	< 0.001
Percentage of nets from campaign source	12.4	88.7	< 0.001

### Household bednet ownership by campaign presence (full sample, n = 256)

**Figure 2:**
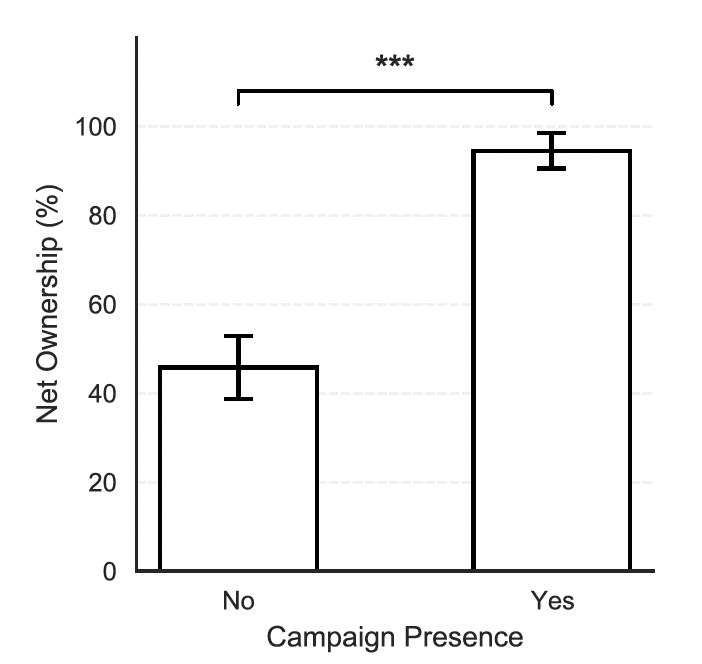
Household ownership (% ± 95% CI) of at least one mosquito net by LLIN distribution campaign status. Statistical significance was determined using a chi-squared test (chi-squared = 78.16, p < 0.001).

Household ownership of mosquito nets and bed coverage differed markedly between regions that had undergone a mosquito net distribution campaign and those that had not. In areas where a campaign had been implemented, 94.5% (95% CI: 90.6%–98.5%, n=128) of households reported owning at least one mosquito net, compared to only 45.8% (95% CI: 38.7%–52.9%, n=190) in non-campaign areas. This difference was highly statistically significant in unadjusted bivariate analysis (χ^2^= 78.16, p < 0.0001; Figure 2), indicating a strong association between campaign presence and household net ownership. Additionally, survey findings confirmed the sources of nets varied considerably between these regions, with a higher proportion of LLINs sourced from clinics and markets in the non-campaign areas (Table 2). These results demonstrate that campaign implementation was strongly associated with a higher proportion of bednets received through free distribution while reducing dependence on routine health facilities or alternative sources. Many homeowners mentioned difficulty attaching the bednets due to high ceilings, a lack of materials such as nails, or their age. A large majority of respondents (77.9%) allowed the surveyor to visually confirm the presence of a mosquito net when asked, while 22.1% declined to do so. This high rate of verification supports the reliability of self-reported bednet ownership in the survey.

**Table 2:** Primary acquisition sources of Long-Lasting Insecticidal Nets (LLINs; n) stratified by campaign presence.

Source of nets	No Campaign (n; %)	Yes Campaign (n; %)
Free distribution	12 (7.5)	342 (83.0)
From clinic	45 (28.1)	38 (9.2)
Purchased	88 (55.0)	22 (5.3)
Other	15 (9.4)	10 (2.5)
Total Nets	160 (100.0)	412 (100.0)

### Household net access (full sample, n = 256)

**Figure 3:**
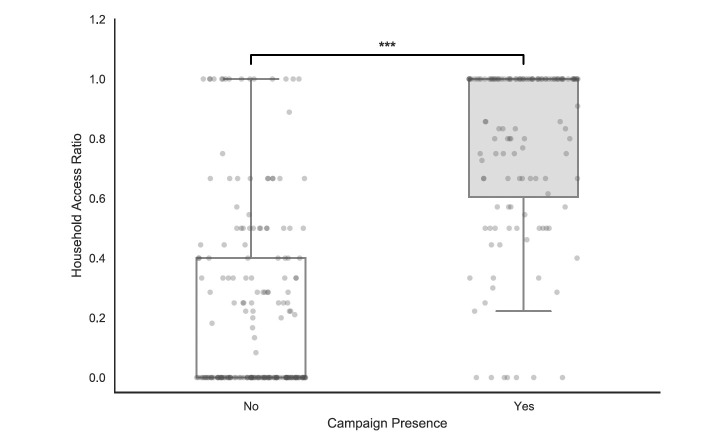
Household LLIN access ratio by campaign status. Household access (defined as the ratio of potential users to residents) was significantly higher in areas reached by a mass distribution campaign (n=128) compared to non-campaign areas (n=187; Mann-Whitney U test, p < 0.001). Horizontal lines within the boxes represent medians, while the boxes and whiskers represent the interquartile range and data spread. Individual data points are displayed as jittered circles representing single households.

The mass distribution campaign was associated with a substantial improvement in household-level bednet availability. Specifically, the intervention was associated with a more than 3-fold increase in the household access ratio, rising from a mean of 0.234 in non-campaign areas to 0.778 in campaign areas (Figure 3). This represents a 232.5% relative increase in potential coverage (p < 0.001), moving the population significantly closer to the international universal coverage target of 0.80.

Household bednet availability was profoundly different depending on campaign exposure, particularly by addressing the burden of total net absence. In areas without a campaign, a majority of households (54.0%) lacked any LLIN access, whereas this 'zero-access' group was reduced to only 5.5% in campaign-exposed areas. Furthermore, the intervention significantly increased the proportion of households achieving the universal coverage benchmark; while only 8.6% of households in non-campaign areas reached or exceeded the 80% access threshold, this figure rose to 61.7% in the campaign group. These data indicate that while the campaign was highly effective at eliminating total coverage gaps, nearly 40% of households in treated areas still fail to meet international targets for universal protection.

### Household net use (cleaned analytical sample, n = 181)

To ensure data integrity and account for fluid household boundaries, bednet utilisation metrics were evaluated within the subset of households meeting strict logical consistency criteria (n = 181 total; n = 115 non-campaign, n = 66 campaign), as detailed in the methods section. By analysing the proportion of residents utilising nets within each household rather than treating individuals as independent observations, this approach inherently controls for intra-household clustering.

**Figure 4:**
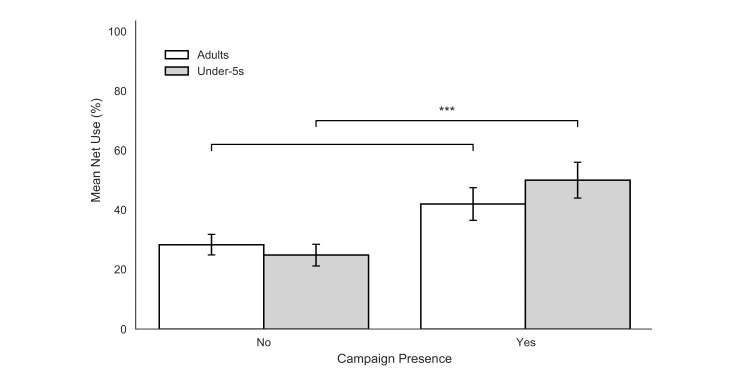
Bednet use among children under five and adults by campaign status. Percentage of children under five and adults who reported sleeping under a mosquito net the previous night in households with and without a recent LLIN distribution campaign. Error bars indicate the standard error of the mean (SEM).

Within this verified analytical sample, the mass distribution campaign was associated with significantly higher household-level utilisation rates among the most vulnerable age cohorts. For households with children under five, the mean within-household percentage of children sleeping under an LLIN doubled, rising from 24.8% (± 3.6 SEM) in non-campaign areas to 50.0% (± 6.0 SEM) in campaign-exposed areas (p < 0.001; Figure 4).

**Figure 5:**
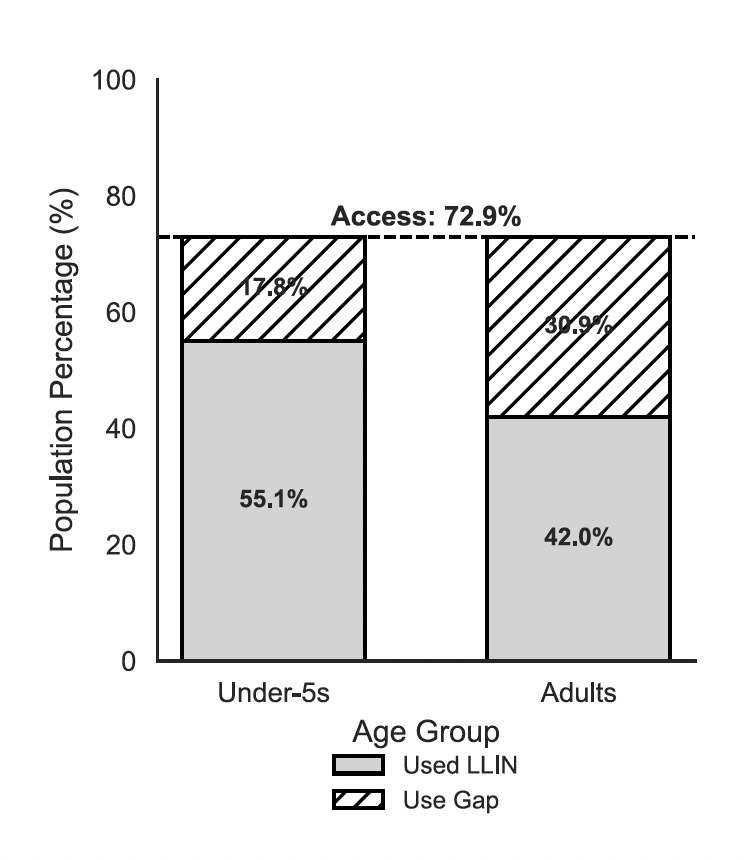
Intra-household LLIN use gap among children and adults with confirmed access. The total height of the bars represents the population access (72.9%) observed in campaign areas. The shaded regions indicate the proportion of the population that slept under an LLIN the previous night, while the hatched regions represent the "use gap"—individuals with household access who did not utilise the net.

While the mean household percentage of adults sleeping under a net also showed an upward trend, increasing from 28.3% (± 3.5 SEM) to 42.0% (± 5.5 SEM), this difference did not reach the threshold for statistical significance (p = 0.052). Notably, in areas without a campaign, adults and children utilised bednets at similar, critically low rates. However, in campaign areas, households demonstrated a clear prioritisation of younger children, with an 8.0 percentage-point gap emerging between child and adult utilisation. Despite increases in ownership, access, and use, there remains a usage gap in both demographics: with a population access of 72.9%, there remains a 17.8% usage gap for children under five, and a 30.9% usage gap for adults to reach their potential usage (Figure 5).

LLIN utilisation among pregnant women (n = 12) was also assessed as a key vulnerable subgroup, though these findings should be interpreted with caution due to the limited sample size. In households exposed to the bednet distribution campaign, coverage levels among adults and pregnant women were similar (42% vs. 41.7%, respectively), suggesting that while children under five were prioritised, a similar utilisation gap may have persisted for other vulnerable adults.

### Impact of distribution campaign on bednet density and deployment (full sample, n = 256)

**Figure 6:**
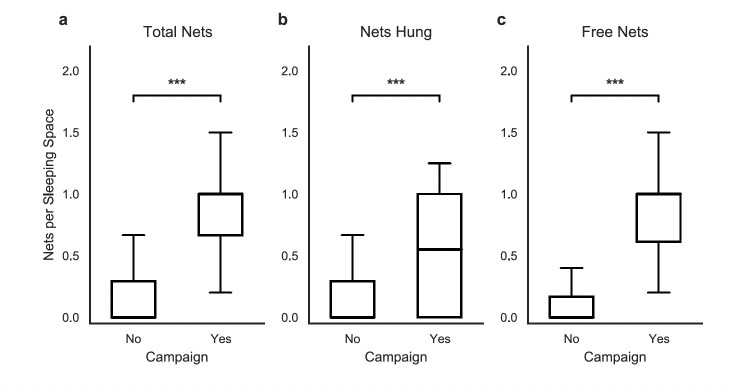
Household bed net availability and installation per bed by LLIN campaign status. (a) Total number of household nets per bed, (b) number of nets actually hung per bed, and (c) number of nets received from the free distribution campaign per bed. Error bars represent standard deviation (SD).

The mass distribution campaign was associated with significantly increased physical net availability and deployment within households. The median total number of nets per sleeping space rose from 0.00 (IQR: 0.00–0.29) in non-campaign areas to 1.00 (IQR: 0.67–1.00) in campaign areas (p < 0.001, Figure 6a). This represents a median increase of 1.00 net per sleeping space, effectively reaching the established target density for basic coverage.

This increase was heavily reflected in the campaign's logistical pipeline; the median number of nets sourced from free distributions increased from 0.00 (IQR: 0.00–0.17) to 1.00 (IQR: 0.61–1.00) per bed (p < 0.001, Figure 6c). Furthermore, the intervention coincided with a significant increase in LLIN installation. The median number of nets hung per sleeping space increased from 0.00 (IQR: 0.00–0.29) to 0.55 (IQR: 0.00–1.00) (p < 0.001, Figure 6b). While this indicates a significant improvement in net hanging behaviour, the gap between the median number of nets owned (1.00) and nets hung (0.55) suggests that physical access alone did not guarantee immediate deployment in all households.

## Discussion

Previous national-level data from the PMI indicated persistent challenges in achieving universal net coverage in Guinea. According to the FY 2023 PMI Malaria Operational Plan, only 55% of households owned any mosquito net, and just 47% had an LLIN. Among households with LLIN, 55% of children under five and 28% of pregnant women reportedly slept under a net the preceding night [1]. These earlier figures highlight the ongoing gap between ownership and utilisation, particularly among high-risk groups, which aligns with the present study’s findings showing suboptimal use even in areas reached by recent LLIN distribution campaigns. Our study built upon this work by specifically investigating the standard ownership, access, and LLIN use indicators based on the presence of a distribution campaign in remote village-level areas in Guinea. However, suboptimal indicators of LLIN use are not limited to Guinea but are found throughout the African continent, with a multilevel analysis across sub-Saharan Africa finding only 32.1% of households with ITN access [20].

While we observed markedly higher net ownership and access in LLIN campaign-exposed areas, highlighting the utility of free mass-distribution pipelines in getting LLINs into residents’ homes, possession does not guarantee sustained utilisation or long-term retention; Koenker *et al.* [21] demonstrated that campaign nets were roughly six times more likely to be given away compared to non-campaign distributed nets within the first few months after distribution. Given that our study examined ownership as soon as two weeks after the campaign’s conclusion, a primary limitation of these data is that they capture the immediate, acute phase of net adoption and initial hang-up behaviour, which may not reflect long-term, stable usage patterns over a multi-year deployment cycle. Future longitudinal work is required to characterise local LLIN attrition, as previous studies have demonstrated the insufficiency of the standard 3-year mass distribution cycle in achieving stable universal coverage [22].

### Deconstructing the "Use Gap": Structural vs. behavioural determinants

Despite the high prevalence of household ownership, there remains a notable observed “use gap” between population-level access and actual bednet use in campaign areas. Optimising future interventions requires distinguishing structural and logistical barriers from behavioural and perceptual factors that limit stable LLIN use.

From a structural standpoint, the observed discrepancy between the household access ratio (0.778) and population access (72.9%) suggests a size-dependent coverage constraint. The relatively lower population access figure may indicate that the fixed-bundle distribution strategy underserved larger households. This suggests that future campaigns in the region should move away from capped distribution (6 nets maximum per household) and toward a strictly needs-based 'one-net-per-two-people' model to ensure equitable protection across varying household sizes. Furthermore, physical installation was heavily hindered by structural and resource constraints; field observations revealed that many households had not hung their nets due to an immediate lack of basic materials like nails or rope, distribution coupons, or the complex suspension geometry required for square-shaped nets. Community members also reported lower-quality nets this campaign cycle with rougher, heat-retaining materials and shorter side lengths rendering it more difficult to tuck into the bedframe.

In contrast, behavioural and social determinants introduced separate, distinct barriers to utilisation. Misconceptions about malaria transmission persist in rural areas, with some respondents attributing the disease to consuming mangos or milk, or exposure to cold weather. These frameworks, combined with a perceived absence of mosquitoes, lower the internal motivation to deploy a bednet. Social desirability bias may have also skewed self-reported reasons for non-use; while households frequently cited external logistical challenges, unprompted qualitative feedback indicated that physical discomfort from ambient heat or the cutaneous burning sensations caused by un-aired, newly treated chemical nets were powerful behavioural deterrents. Additionally, social patterns such as crowded and fluid sleeping arrangements, shared multi-generational beds, and the temporary relocation of nets to agricultural fields for crop protection further drive down domestic usage. Although community health workers (CHWs) are formally tasked with ensuring that LLINs are properly installed post-campaign, there is currently no coordinated oversight or supervision, and many do not fulfil this responsibility. Previous work has demonstrated that the non-use of LLINs had a greater impact on overall intervention effectiveness than physical degradation or insecticide loss [23]. These findings highlight that getting LLINs into residences is merely a prerequisite; greater emphasis must be placed on behaviour change communication (BCC) and post-distribution follow-up, mediated through structured CHW systems, to ensure that distributed nets are installed and used.

### Methodological limitations

When interpreting these findings, several methodological limitations must be explicitly considered, and these cross-sectional data should be viewed as exploratory associations rather than definitive evidence of causal inference.

First, household selection within the villages relied on convenience sampling based on real-time availability during daytime field visits, introducing potential selection bias by over-representing households where residents were more frequently home and easily accessible. However, because data collection was distributed across more than thirty distinct villages, the multi-site nature of the study helps capture diverse community contexts, potentially partially mitigating localised biases.

Second, our unadjusted bivariate analyses do not account for unmeasured confounding factors such as socioeconomic status, household head education, or precise proximity to routine health facilities which may independently influence both campaign reach and health-seeking behaviours. We deliberately avoided multivariable modelling because our modest sample sizes risked overfitting, and field realities in rural Guinea, such as approximated ages, recall bias in education, and occupational multiplicity, made these socio-demographic variables highly prone to classification error. A conservative, non-parametric bivariate approach was therefore chosen to ensure a focused analysis of group differences.

Additionally, data integrity required excluding 75 households that failed logical consistency checks for the utilisation analysis, leaving an imbalanced utilisation sample (n = 115 non-campaign vs. n = 66 campaign). While necessary to mitigate measurement error from fluid household boundaries, this substantial data exclusion limits sample size and introduces potential sampling bias; thus, these findings represent conservative associations rather than definitive causal impacts.

Finally, data collection took place entirely during the rainy season, a period of peak malaria transmission and high ambient mosquito density. Consequently, the observed usage rates likely reflect a seasonal peak and over-represent annual net utilisation. During the dry season, a diminished perception of malaria risk combined with increased indoor temperatures often exacerbates physical discomfort, leading to sharp seasonal drops in net compliance. Because these surveys occurred as soon as two weeks post-campaign, these figures may not capture stable, long-term usage patterns, as households frequently experience an initial adjustment period during which logistical barriers or behavioural delays in physically hanging new nets can temporarily artificially depress utilisation rates.

### Implications for global health equity

Limited access to health care remains a major challenge in Guinea, with the Ministry of Health estimating that only 55% of the population can readily reach health services [3]. Given that prenatal consultation rates (in which there is routine LLIN distribution) and facility-based deliveries remain low in Guinea, increasing access to and utilisation of these services may concurrently enhance LLIN uptake from health centres. The relatively low coverage of adults and pregnant women sleeping under LLINs in campaign areas (42.0% and 41.7%, respectively) underscores the need for intensified behavioural change communication and community sensitisation to bridge the 'ownership-to-use' gap. Although the small sample size of pregnant individuals in this study (n=12) precludes broad generalisation, the observed parity between general adult and maternal utilisation rates warrants further investigation into the specific barriers facing high-risk groups in rural Guinea.

In conclusion, while this demonstrates that LLIN distribution campaigns are strongly associated with increased household ownership, access, and bednet use in children under five, substantial operational gaps remain. Recent budget reductions and funding uncertainties affecting major donors such as USAID, the Global Fund, and PMI may jeopardise the continuity of bednet distribution campaigns in Guinea. Moreover, indoor residual spraying (IRS) coverage remains limited in Guinea, with malaria prevention efforts continuing to rely primarily on LLIN distribution campaigns. The low adoption of complementary interventions such as IRS underscores the importance of maintaining and expanding net-based strategies. As opposed to current three-year distribution cycles, continuous distribution strategies could enhance efforts to reach universal coverage [22]. Effectively expanding such strategies will require future campaigns to address the structural and social determinants that impede bednet usage in households. Given the significant differences in household LLIN indicators observed between campaign and non-campaign areas in this study, sustained financial and logistical support remains essential to prevent backsliding in malaria control efforts. Ultimately, ensuring that the most remote populations, who bear the highest burden of disease and have the least access to clinical care, are not left behind by funding shifts is a matter of global health equity.

## Conclusions

This study provides cross-sectional evidence that LLIN distribution campaigns are associated with markedly higher household net ownership, access, and use among children under five within the surveyed communities. Despite these observed increases, practical barriers, misconceptions, and suboptimal net use persist, underscoring the need for complementary behaviour change interventions and post-campaign structural support. When interpreting these outcomes, important methodological limitations must be considered, including our reliance on convenience sampling, self-reported utilisation metrics prone to social desirability bias, and a substantial data exclusion process that removed logically inconsistent household surveys. Furthermore, because this evaluation excluded communities across the Upper Guinea and Forested Guinea regions, these specific parameters may not be broadly generalisable across the entirety of the national landscape. Nevertheless, these findings offer valuable contextual insights for designing LLIN campaigns in similar low-resource, malaria-endemic settings, emphasising that strategic needs-based distribution and targeted community engagement are critical to achieving high coverage and reducing malaria transmission vulnerabilities. Future efforts should focus on addressing both material and socio-behavioural local barriers in partnership with local community health workers.

## Data Availability

All survey instruments, anonymised data, and analysis scripts used in this study are available in a public GitHub repository: https://github.com/emretoner/Guinea_BedNetStudy. The repository includes the survey forms (both Google Forms and printable versions), raw and cleaned datasets, and the Python code used for statistical analysis and figure creation.
